# An organizational analysis of road traffic crash prevention to explain the difficulties of a national program in a low income country

**DOI:** 10.1186/s13104-015-1472-6

**Published:** 2015-09-28

**Authors:** Tania Vogel, Daniel Reinharz, Marissa Gripenberg, Hubert Barennes

**Affiliations:** Institut Francophone de Medecine Tropicale, Vientiane, Lao PDR; Département de médecine sociale et préventive, Université Laval, Laval, QC Canada; Epidemiology Unit, Pasteur Institute, Phnom Penh, Cambodia; INSERM, ISPED, Centre INSERM U897-Epidemiologie-Biostatistique, 33000 Bordeaux, France; Agence Nationale de Recherche sur le VIH et Hépatite, ANRS, Phnom Penh, Cambodia

**Keywords:** Road traffic crash, Road traffic accident, Prevention, Injuries, Helmets, Head trauma, Mortality, Organizational analysis, Lao People’s Democratic Republic, Laos

## Abstract

**Background:**

Road traffic crashes (RTC), that daily kill 3400 people and leave 15,000 with a permanent disability could be prevented through the implementation of safety programs developed in partnership with governments and institutions. The relationship between key stakeholders can be a crucial determinant to the effectiveness of road safety programs. This issue has rarely been addressed. We conducted a detailed organizational analysis of the stakeholders involved in road safety programs in Lao People’s Democratic Republic (Lao PDR).

**Methods:**

A case study was performed. The framework used was a snowball effect in which the characterization of all key stakeholders and the links between them, as well as the factors that led to these links, were determined. The effect of the relations between key stakeholders on the prevention of RTC was assessed through an analysis of the transactional, intangible and controlling factors that influence these relationships.

**Results:**

The design and implementation of road safety programs in Lao PDR suffer from weak relationships between stakeholders and a poorly functional bicephal leadership between the Ministry of Public Works and Transport and the non-governmental organisation called Handicap International. This poor coordination between key stakeholders is evident, particularly in the area of collective action and is reinforced by a lack of interest from several different stakeholders. Most agencies do not prioritize road safety. Uneven distribution of funding is another contributing factor. Strengthening the leadership is crucial to the success of the program. Some organisations have skills, power the decision making and the allocation of resources in regards to road safety programs. Encouraging participation of these organizations through a more prominent position would thus result in a better collaboration. Non-monetary rewards would further help to strengthen collaborative work.

**Conclusion:**

The bicephal nature of the leadership of road safety programs proves detrimental, is associated with a weak coalition between stakeholders, and contributes to the declaimed poor effectiveness of the existing programs. The study has identified non-monetary and realistic means of strengthening the collaboration between key stakeholders. Stakeholders need to revise their interpretive schemes, in order to actively support the reinforcement of government leadership of road safety policies.

## Introduction

Worldwide, road traffic crashes (RTC) kill 1.24 million people each year leaving 5500–13,700 with a permanent disability [1]. The situation is particularly alarming in low and middle income countries (LMIC) where over 90 % of road traffic fatalities occur. The highest death rates are found in the African and South-East Asia Region (each year 24.1 per 100,000 and 18.5 per 100,000 population, respectively). Road traffic injuries (RTIs) are the leading cause of death among individuals in the economically productive age range of 15–44 years and the second leading cause of death among 5- to 14-year-old in the world [2]. The impact on disability-adjusted life years (DALYs) is considerable: nearly 4.5 % [3].

Yet, RTC and their consequences, i.e. deaths and disabilities, could be reduced through preventive programs. Such programs are typically composed of the implementation of public policies on crash prevention. These include the development of safer routes and stricter enforcement of regulations resulting from these policies, as well as public awareness interventions. According to the latest WHO report, only 28 countries, covering 7 % of the world’s population, have comprehensive road safety laws on five key risk factors: drinking and driving, speeding, failure to use motorcycle helmets, seat-belts, and child restraints [1].

Many of these procedures are formally established in LMIC but their implementation remains weak [4]. Because of the impact of RTC both at a population and at an individual level, it is important to evaluate such programs from different angles in order to better identify how to improve their effectiveness and efficiency.

Organizational analysis allows for the understanding of the complexity and structure of these programs. An organizational analysis is a process by which an organization’s systems, capacity, and functionality are assessed in order to increase its efficiency, performance, and output [5]. This has particularly been used in the healthcare sector to determine if the goals set out by the organisation are met, what needs to be improved and what are the strengths of the different departments or organisations [6, 7]. The main interest here is the dynamics between all actors concerned, i.e. factors that support or hinder the collaboration between groups and organizations. Often actors belong to different sectors. A coordinated mobilization is considered a determinant of the effectiveness and efficacy of complex preventive programs. The reasoning behind the choice of this type of analysis for RTI’s is the ability of organisational analysis to map out and define the level of involvement of each organisation as well as their level of capacity to be involved. Organizational analyses can provide useful information on the interpretation of statistical data [8]. It can show how programs are implemented and how they function in real life.

Lao People’s Democratic Republic (Lao PDR) is a LMIC. Lao PDR, is now facing a rapid increase in RTIs associated with its economic growth [9, 10]. Here, an estimated 1050 deaths occur each year due to RTIs [11]. Perspectives are alarming considering the increase in the number of vehicles foreseen in the future for this part of the world [9–11]. Most often fatalities and RTI affect motorcyclists (84 %), in particular young drivers (17–25 years of age) on Friday and Saturday nights [9, 10]. The presence of alcohol and amphetamines are frequently associated with these crashes [10]. Hospital surveys in the capital Vientiane show that head injuries are common (42 %) among motorcyclists; less than 30 % of them wore helmets and 42 % had consumed alcohol [9, 12].

To face the increasing burden of RTC in Southeast Asia, the Association of Southeast Asian Nations (ASEAN) added components to improve transportation in its 10 years (2005–2015) development plan [13, 14]. The ASEAN plan specifically outlines the increase in the knowledge of road safety programs and how to expand and build more modes of transportation. ASEAN assisted Lao PDR in building its own action plan for road safety. In addition, in Vientiane, the capital of the Lao PDR, a maintenance committee was established in 2001 to ensure that streets and trucks are well maintained [15]. Technical maintenance of cars has also become mandatory every 2 years. In 2002, a law on compulsory helmet wear was passed, although limited to drivers only, despite evidence that helmets could reduce fatalities by 41 % among passengers [16]. This law is enforced by the police [17].

The plan elaborated by the Lao PDR government focuses on education, training, evaluation of buildings and the environment, as well as emergency services and research and assessment [13]. With this plan, the Lao government hopes to reduce the mortality rate from eight deaths by 10,000 vehicles in 2010 to five in 2015 and two in 2020 [14]. It is feared that the trend observed in 2005 of a 12 % yearly increase in road crash, has however not decreased [10, 17].

The absence of or a poor relationship between key stakeholders can be a crucial determinant limiting the effectiveness of road safety programs but this issue has rarely been addressed. We analysed the nature of relationships and the coalition between the organisations in charge of preventing RTC in Lao PDR. We evaluated the strength of coalitions and how much control and power they have.

## Method

### Study procedure

A case study was performed based on a conceptual framework [8, 18–21] used in two former studies [6, 7]. This framework is based on a hypothesis. When stakeholders (organisations that have the potential either financially or by knowledge to collaborate on road safety prevention) collaborate (form a *coalition*), the effectiveness and efficiency of their common goal are amplified. Indeed, actors are constrained by the structure in which they evolve, as well as by the interpretive schemes (which are a way of analysing and defining the strength of the ties between two different organisations) that define morally and cognitively what can be done, both elements influencing one another. In other words, the framework allows for the description of the state of the *coalition* between stakeholders. Coalitions can be defined as formal and/or informal alliances between stakeholders following a leader, to more effectively reach a common objective: in this case, the reduction of RTC. *Actor*(*s*) refer to individuals belonging to the same organization (professional and/or institutional) who have the capacity to act [20].

This framework allows for the identification of all involved actors, the characterization of the *relations* between them and the identification of factors that led to these relations, i.e. the dialectic between the organizational structure and the interpretive schemes [19]. In our analysis we looked at three different types of the aforementioned relations: (1) transactional, i.e. tangible reasons for collaborating: the assets each actor can bring to the coalition and the expected benefits sought from participating in a common project, (2) intangible, i.e. friendships or shared ideologies and (3) controlling, i.e. the capacity of a group to impose the participation of others [18].

To better visualize the coalition, this framework was complemented by a diagram of the interrelationships between the organizations concerned, based on two dimensions: their involvement in the prevention of road crashes and the limits involved to support an eventual involvement.

Links were estimated as weak if one the following situations occurred: (1) occasional contact between two organizations (less than once every few months or (2) road safety was not a main topic or barely discussed, or (3) outdated and inexistent current exchange between organisations despite links in the past.

Links were defined as strong if (1) formal and regular meetings were taking place with road safety programs acting as the main point of discussions; or (2) regular communication, discussion or development or implementation of road safety projects together; or (3) when strengths of each actor were used to complement the weakness of others.

Two sources of information were used: (1) all documents in French or English, related to road safety in the Lao PDR retrieved from online document and peer reviewed journals; (2) semi-structured interviews that were conducted with key actors working in the capital of Vientiane.

One of the researchers (TV) performed the interviews face to face. Organisations were asked to select one representative speaker, designated to answer the questions on their behalf. Informants had to have a formal involvement in their organization within the road safety topic or department. The study was a qualitative study and the sampling did not aim to represent the general target population, but to reflect the heterogeneity of the organizations concerned by the problem [22]. Most interviews were at the management level, involved in extensive decision making such as how the budget is spent, how they would intervene etc.

A snowball procedure was used to identify potential participants and organisations. The first person and organisation that were targeted were contacts provided by the research institute where the project took place. During each interview participants were asked for any contact, people or organisation they recommend to meet. New participants were included until the investigators were convinced that the information was saturated [21, 22]. Individual interviews, with an average length of 45 min, were conducted in English (four interviews) or French (nine interviews), with all participants being comfortable with one or the other language. A validated interview guide composed of themes related to the dimensions of the conceptual framework was used [20].

The following topics were addressed during the interviews: (1) the opinion and experience of the organisation on the implementation, challenges, barriers and results of the road safety programs in the country, (2) weaknesses, strengths and suggestions for improvement, (3) the main reasons contributing to the current situation and (4) resources and time that the organisation planned or put forward in the prevention program.

When an organisation responded negatively to one question, the researcher would question if the organisation was willing to help the situation and to describe the limits and/or barriers to their participation. The characteristics of the relationship with the other organisations and the leading organisations were also addressed.

Interviews were recorded and transcribed immediately after the interview was finished.

### Study analysis

Analysis took place concomitantly with data collection. Each interview was dissected and the appropriate information was categorized according to the resources or the power to create a change that a particular organisation had compared to the involvement this organisation had in relation to road safety. Coding was based on the conceptual framework of the study and done for each interview before conducting the next encounter. Transversal analysis was performed after each interview. It allowed determining the strengths between the links that existed between organisations. This transversal analysis was conducted to identify re-occurring themes between each of the different interviews conducted. Once a re-occurring theme was discovered, it was flagged for further investigation in prospective interviews. Answers and items were grouped in themes, and these themes were eventually explored within the other organisations via the remaining interviews. This was made possible within this framework as the analysis of each interview was done before the following interview was scheduled. Hence, themes were able to be explored more in-depth with some of the organisations.

Another component to the analysis was the creation of two diagrams. The placement of the different organisations on the diagrams was determined by comparing control, resources, transactions and road safety as a priority.

Validation of the results was based on three components: (1) the triangulation of information, (2) the fact that two investigators performed the analysis independently (when a discrepancy occurred in the coding of the data—i.e. when investigators placed a data item in two different categories, consensus was sought); (3) sending a preliminary report to informants in order to make sure that their organization was well represented in the analysis [23]. This type of validation was used to avoid bias caused by any potential language barriers. It was used as a way to ensure that all the information was received and analysed properly, since both researchers did a first validation alone then compared. This validation method was also used in order to make sure enough appropriate information was collected during the interviews in order to answer the questions.

A French report was written and distributed to participants as the prominent language of the agencies was French. This allowed for them to provide some initial comments. A report was then written in the context of a master’s project submitted to QC, Canada where French is the main language.

### Ethics statement

Ethical approval was granted by the Lao Medical Ethics Committee. Interviewees were informed about the study and included after they provided written informed consent. Confidentiality was guaranteed and great care was given to interviews taking place in privacy. Finally, assurance was given that it would be impossible in the report and articles to identify who raised the points that emerged in the analysis.

## Results

There were no refusals to participate in the study. Thirteen interviews were performed in total. Informants came from the following organizations: Ministry of Public Work and Transport (MPWT), Ministry of Health, Handicap International (HI), Mitthaphab Hospital (where the trauma department is located), Town Hall of Vientiane, Lao Red-Cross, French Red-Cross, WHO and UNICEF.

**Fig. 1:**
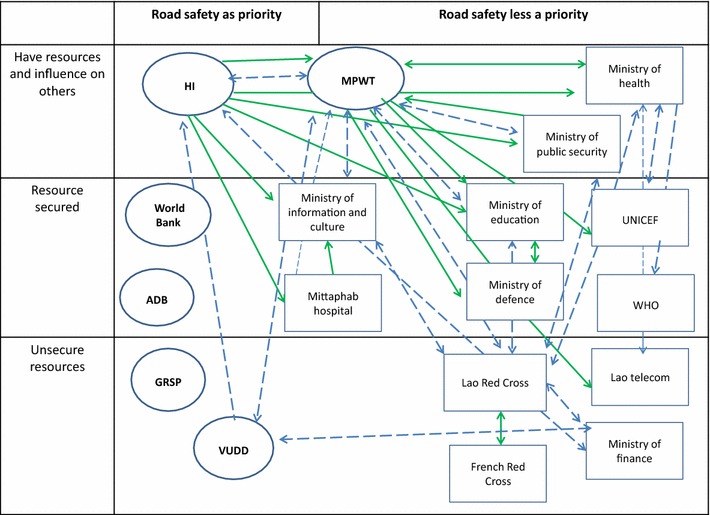
Relationship between actors concerned by traffic safety in Lao PDR, control and transactional links. *ADB* Asian Development Bank, *MPWT* Ministry of Public Work and Transport, *GRSP* global road safety partnership, *HI* Handicap International, *GRSP VUDD* Vientiane urban development department. Control links *continuous green arrow*. Transactional link *discontinuous* *blue arrow*

**Fig. 2:**
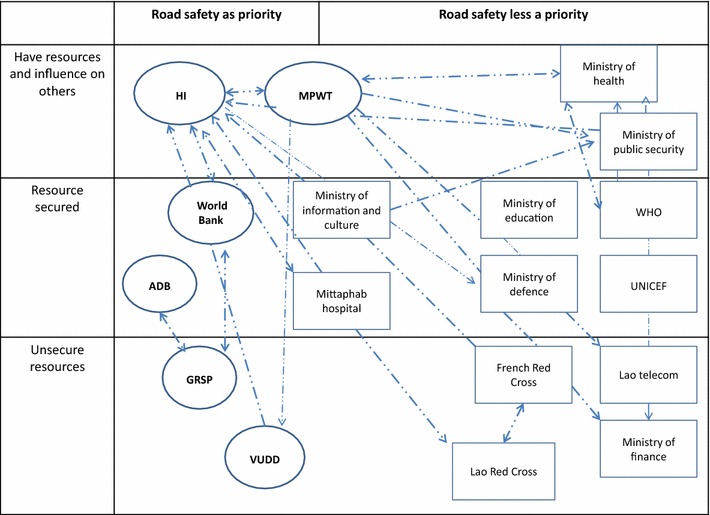
Relationship between actors concerned by traffic safety in Lao PDR, intangible links. *ADB* Asian Development Bank, *MPWT* Ministry of Public Work and Transport, *GRSP* global road safety partnership, *HI* Handicap International, *GRSP VUDD* Vientiane urban development department. Intangible link *discontinuous* *dots* and *line blue arrow*

Figures 1 and 2 show the actors with their relationships (control and transactional, Fig. 1; intangible links Fig. 2) on a grid that also presents the priority they give to road safety activities, and their capacity to act, i.e. presence of resources, human, material and financial, devoted to road safety activities.

Actors concerned with road safety form a weak coalition, i.e. they collaborate poorly on road safety activities. This loose coalition is based on a bicephal leadership constituting of the ministry (MPWT) and HI, a foreign nongovernmental organization (NGO). This bicephal leadership emerges due to the fact that neither of these actors detains the two kinds of assets deemed necessary for a unique leadership to form: expertise to develop programs and legitimacy to implement them. The NGO was the initiator of road safety prevention programs in the Lao PDR, as it has the expertise and the funds required. Its main interest in building a coalition comes from the necessity to legitimize its programs, and the need to secure the involvement of other sectors to tackle the diverse dimensions attached to an effective program. The ministry has the legitimacy to allow projects that affect road safety. It has a road safety department and a budget for prevention activities for road crashes. However, it lacks expertise and recognizes that many activities needed to strengthen its capacity for action in the promotion of road safety, are under the responsibility of other sectors over which it has no control.

The other actors can broadly be categorized into two groups: organizations with a clear implication on safety activities, expressed as activities on road safety stated among specifications of the job sets, and those whose involvement is mainly limited to participating in meetings.

Links between these organizations are of three types considered in the theoretical framework (tangible, intangible and controlling). Several organizations are linked through these three types concomitantly, showing that beyond administrative imposition, personal and utilitarian considerations also prevail. Links are weak especially between those who do not prioritize road safety among their activities. The links between the MPWT and its partners are rather formal, as most are obligations imposed by the government. The links between HI and its partners are more of the transactional type. In fact, HI needs to find or use some skills and assets (in fields that he does not possess) with his partners.

The fact that the coalition was loose is due to, according to the respondents, factors related to both the interpretive schemes and to the organizational structure. On the interpretive scheme side, most actors admitted that they did not have the expertise required and above all, that they did not think it was their responsibility to prioritize road safety activities. This was notably the answer of two agencies of the United Nations; WHO and UNICEF which both have the expertise and financial means to support road safety activities. Both of them evoke a lack of solicitation to justify their poor participation in this field.

On the structural side, the main factors that emerged as associated with a poor coalition were hierarchy and funding. The hierarchical nature of the Lao system was seen as a constraint for the effectiveness of the programs when they are led by a bicephal leadership. A leadership divided with one head having the expertise and the funds, and the other one the legitimacy to impose programs, is seen as poorly effective, as no one can be confident about who detains the authority to impose or incite his participation.

Finally, lack of funding is seen by many as the main hindrance for convincing actors to get involved in road safety activities. Many respondents complained that nearly all funds channelled by donors for road safety activities are directed to two ministries MPWT and Ministry of Public Security, the major beneficiaries that have the responsibility to enforce traffic laws. Other ministries are left with little monetary incentives to act.

The major consequence of this poorly knitted network of potential collaborators, which is effectively declaimed by all respondents, is the partial implementation of the 2005 plan of action on road safety, elaborated under the auspice of ASEAN. This plan was built with the involvement of several ministries (MPWT, education, finance, health, security, information and culture) and foresees the production by each of them of their own prevention program. Yet, most of them have not yet produced any documents.

## Discussion

The study highlights the links between different stakeholders for the implementation of road traffic injury prevention. It illustrates that the coalition between stakeholders is weak, and that this weakness is a main determinant of the declaimed poor effectiveness of the existing prevention programs. The bicephal nature of the leadership was seen as problematic by most of the stakeholders. A reassessment of the leadership might be regarded as a central issue in order to build a stronger coalition. This bicephal nature is based and justified by a necessary complementation of assets. A stronger leadership by the public head would reinforce all types of links in this environment that is made of a complex web of inter-sector cooperation: transactional, intangible and controlling. A strong public leader is seen as someone who has the legitimacy to define which assets the diverse actors should possess such as; ability to commit to the implementation of a project or the availability of resources (time, monetary etc.) to dedicate to a project. This would allow for a better distribution of the responsibilities that are expected from each actor involved.

Incentives are clearly the cornerstone of the capacity to mobilize stakeholders. Financial incentives would build or reinforce transactional links. However, non-financial incentives might also be effective, as the setting in Lao PDR is favourable for intangible and controlling links. For example HI does not have very much power in decision making or resource allocation. Therefore allowing a government organisation that has this power, such as the MPWT, to control and make decisions based on resource allocation can give them an incentive to participate and be involved in the ongoing project. A stronger leadership by the public leader might be able to enforce these non-financial incentives too. This would provide the impetus for sharing expertise between stakeholders, for example between different Red-Cross organizations, or one of the Red-Cross organizations and WHO. Sharing expertise would benefit those who feel poorly equipped to participate in a common project.

The role of a public leadership has rarely been studied in LMIC, although it emerges as one of the main recommendations in the 2009 WHO report on road safety [4]. Clearly, our results empirically support one of the key WHO primary recommendations that it is the role of the government to ensure that the roads are safer. Measures should be taken to encourage the government to become the leader in this matter.

Extensive research has been dedicated to factors that are necessary for the success of RTC prevention programs. One of the most noteworthy efforts to reduce RTCs is the RT-10 project [24]. Established in 2010, this multi-country program funded by the Bloomberg Philantrophies was set to reduce global burden of RTCs, within 5 years. Based on high level working groups of stakeholders, local ministry of health and organizations such as WHO and the Global Bank road safety facility, the project set national specific work plans. With the evaluation of the project set to take place this year, projected results of the project look promising and cost effective with up to 10,000 saved lives [25]. Other studies have highlighted the importance of targeting underlying, country specific risk factors for RTC such as drink driving, over-speeding and lack of seatbelt or helmet use. As such, a better distribution of task between stakeholders might improve their motivation in participating in the program in Lao PDR.

Finally, the study suggests that in a context of Laos PDR a strong governmental involvement, led by for example by the Ministry of Health, together with key stakeholders such as WHO and HI, focusing on the country specific risk factors and an adequate surveillance of the program will lead to the most cost effective and effective RTC prevention programs in Laos.

### Limitations

This study has several limitations. The results of this study should be interpreted while taking into consideration the limitations related to the conduction of a qualitative study performed in Lao PDR by non-Lao investigators. Although all respondents were fluent in English or French, for most, these languages are not their mother tongue. This could have in some way limited the transmission of information, although the openness of the interviews makes it quite unlikely.

During the interview selection a snowball technique was used. This can result in missing some small NGO’s working in isolation or not well known in the field in the main cities. This bias was avoided mostly by an attempt to contact other NGO’s in the area, research of the country’s road safety programs as well as interviewing a small organisation in the North of the country which is more isolated [10]. Another limitation is the use of a recording device to collect data during the interview that does not take into account body language nor the conversations that may have happened after the recorder was off. However face to face interviews and a careful transcription probably limited this issue. While the snowball technique was a great way to gather and learn about new interview candidates it also led to a specific type of candidates that were being interviewed. Most interviewees recommended someone of the same level as them from a similar organisation. Heterogeneity was harder to achieve because of this and may have caused a slight limitation.

Another main limitation is the potential public face the interviewee is putting on. It was difficult to know what is being told to please the public compared to what the reality is of the organisation. In other word, an organisation may have road safety as a program target but stakeholders may know within the reality of their organisation that their goal will not be achieved. It was hard to differentiate what was an attainable goal by the organisations and what was not. Although great care was given to the validity of the data, through triangulation, coding by two investigators and returning the report to interviewed individuals, one cannot exclude some strategic (by informants) and interpretation (by analyzers) bias [22].

## Conclusion

Road safety in the Lao PDR is a public health problem firmly based on the difficulty to lead all involved actors to participate in a collective action. Bicephal leadership and weak relationship between major stakeholders proved detrimental to the program as well as an uneven distribution of funding and tasks. This organizational portrait suggests that key actors such as WHO, should be advised to revise their interpretive schemes, in order to actively support the reinforcement of government leadership on road safety policies. Efforts to involve key stakeholders to help the government become an effective leader in this field should increase the effectiveness of the programs, without the need to invest additional resources.

## Abbreviations

RTC: road traffic crashes
LMIC: low–middle income country
RTI: road traffic injuries
DALY: disability adjusted life years
WHO: World Health Organisation
Lao PDR: Lao People’s Democratic Republic
ASEAN: Association of Southeast Asian Nations
MPWT: Ministry of Public Works and Transport
HI: Handicap International
NGO: non-governmental organisation
